# A Nerve of McKenzie With a Variant Communicating Branch Between the Vagus Nerve and Cranial Root of the Accessory Nerve: A Cadaveric Case Report

**DOI:** 10.7759/cureus.14343

**Published:** 2021-04-07

**Authors:** Katie Carsky, Joe Iwanaga, McKenzie Clark, Aimee Aysenne, R. Shane Tubbs

**Affiliations:** 1 Department of Neurosurgery, Tulane University School of Medicine, New Orleans, USA; 2 College of Human Sciences, Florida State University College of Medicine, Tallahassee, USA

**Keywords:** nerve of mckenzie, variant, anatomy, craniocervical junction, neck surgery

## Abstract

Anatomical variations of the craniocervical junction including a nerve of McKenzie, a branch between the spinal accessory nerve (XI) and the ventral root of the first cervical nerve (C1), have been identified. During routine dissection, a nerve of McKenzie with an interneural connection between the cranial root of the accessory nerve and the vagus nerve was observed on the left side. To our knowledge, a case with these two anatomical variations in the same cadaver and on the same side has not previously been reported. These variants may complicate surgery of the nerves of the craniocervical junction, and should thus be appreciated by the surgeon. Here, we discuss this case, its possible embryological origins, and the clinical significance.

## Introduction

The nerve of McKenzie is a communicating branch that can be found between the spinal accessory nerve (XI) and the ventral root of the first cervical nerve (C1). It may pierce the first denticulate ligament [1] or course anterior to this ligament [2]. Identification of the nerve may become important in the setting of intradural rhizotomy for spasmodic torticollis [1].

We discovered a variant nerve of McKenzie in which connections also existed between the cranial root of the accessory nerve and the vagus nerve. This case highlights the existence of variant interneural connections in the form of a nerve of McKenzie, including but not limited to the connection identified. We present this case for neurosurgeons, and head and neck surgeons to better understand the existing variants found simultaneously with a nerve of McKenzie.

## Case presentation

**Figure 1:**
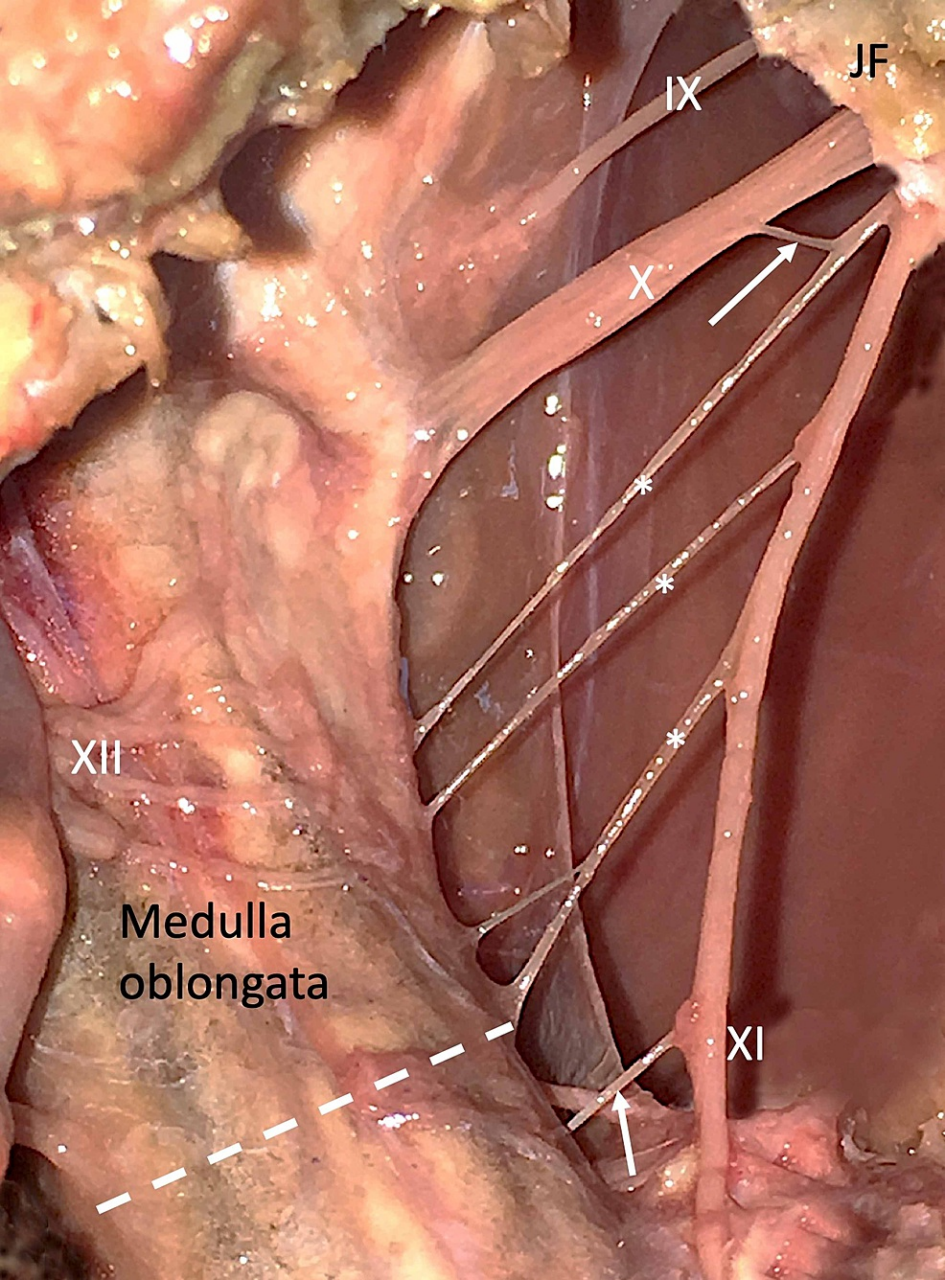
Anterior view of the left craniocervical junction. The hypoglossal nerve (XII) rootlets are transected distally and folded medially. Asterisk notes the cranial roots of the accessory nerve (XI). The uppermost cranial root has a communicating branch (upper arrow) joining the vagus nerve (X). A nerve of McKenzie (lower arrow) is also seen joining XI. Asterisk: Cranial roots of the accessory nerve. FM: Foramen magnum JF: Jugular foramen X: Vagus nerve XI: Accessory nerve XII: Hypoglossal nerve

During the anterior dissection of the skull base in a fresh-frozen, 84-years-old at death male cadaver, a nerve of McKenzie with an interneural connection between the cranial root of the accessory nerve and the vagus nerve was observed in the craniocervical junction on the left side (Figure 1).

The neural connection traveled from superomedial to inferolateral in an oblique manner. The specimen was 84 years old at death. No other anatomical variations were identified in the regions dissected. Additionally, no nerve of McKenzie or connections between the vagus and cranial root of the accessory nerve was found on the right side of this specimen. The authors sincerely thank those who donated their bodies to science so that anatomical research could be performed. Results from such research can potentially increase mankind’s overall knowledge that can then improve patient care. Therefore, these donors and their families deserve our highest gratitude [3].

## Discussion

The nerve of McKenzie has previously been reported with varying frequencies, with McKenzie [2] himself reporting a frequency of 50% and the most recent study reporting that the nerve was found on 70% of sides [1]. The branch typically courses over the anterior spinal artery and vertebral artery posteriorly before either passing anteriorly to the denticulate ligament [4] or piercing the first denticulate ligament [1]. 

Embryologically, the accessory nerve and the vagus nerve are of shared origin. Both nerves arise from the same ganglionic crest, which lateralizes at roughly 20 days of development. The accessory nerve and the vagus nerve are separated around week four during normal development [5]. 

By week four of development, the caudal end of the neural tube begins to form the spinal cord, and the rhombencephalon gives rise to the myelencephalon after five weeks. The myelencephalon then forms the medulla oblongata [6], where both the vagus and accessory nerves stem from.

Variations of the accessory nerve including duplicate nerve and direct connection with the facial nerve have been previously described [7]. A communicating branch between the dorsal root of the first cervical nerve and the vagus nerve has also been described in Bactrian camels during the investigation of the craniocervical junction [8]. Few connections between the spinal accessory nerve and vagus nerve at the level of the jugular foramen have been previously reported [9], similar to the described case. Knowledge of variants of the accessory nerve and vagus nerve anatomy adds to our current understanding of the nerves of the craniocervical junction and may be of clinical value. 

The literature suggests that a typical nerve of McKenzie in which communication exists between the spinal accessory nerve (XI) and the ventral root of the first cervical nerve (C1) becomes clinically significant in the setting of the intradural selective rhizotomy, a treatment for spasmodic torticollis. As the ventral root of a spinal nerve should contain motor fibers, a contribution from the C1 nerve to the spinal accessory nerve should contain motor fibers that might also innervate the sternocleidomastoid and trapezius muscles with the rest of the spinal accessory nerve. Moreover, the craniocervical junction is a transitional area between occipital and cervical somites and cranial nerve nuclei. Therefore, a mixture of somatic and pharyngeal arch-derived tissues is found in this unique area. Such mixing of tissue types adds support to the findings, whether functional or embryological, that a cervical spinal nerve branch i.e., the nerve of McKenzie of C1 to spinal accessory nerve communications.

Some believe the nerve of McKenzie should be sectioned during operation for spasmodic torticollis [1,4]. However, this might result in weakness of the motor function of the trapezius and sternocleidomastoid muscles. This suggests that awareness of variant nerves of McKenzie is important for clinicians.

## Conclusions

This case highlights a variant nerve of McKenzie in which communication between the accessory and vagus nerves exists. Knowledge of this possible anatomy may be clinically significant, and is, therefore, important for neurosurgeons, orthopedic surgeons, and head and neck surgeons to be aware of.
